# Empowering and Motivating Undergraduate Students Through the Process of Developing Publishable Research

**DOI:** 10.3389/fpsyg.2019.01007

**Published:** 2019-05-03

**Authors:** Sue K. Adams

**Affiliations:** Human Development and Family Studies, University of Rhode Island, Kingston, RI, United States

**Keywords:** publishing, undergraduate, research, mentoring, social science, psychology

Mentoring undergraduates in research is a truly rewarding endeavor. There are immense benefits for both students and faculty mentors who engage in high-quality undergraduate research mentorship (Bowman and Stage, 2002; Osborn and Karukstis, 2009). For students, the experience allows them to expand their skills and knowledge, increase self-efficacy and self-confidence, increase learning gains, and connect classroom learning to real-world settings (Palmer et al., 2015). Becoming part of a research lab can inspire future graduate studies or job paths in a certain field, and provide a competitive edge over peers (Shellito et al., 2001; Davis and Jones, 2017). For faculty, mentorship can promote the transfer of academic “DNA” and generate meaningful scholarship (Lancy, 2003). The focus of this paper is to discuss principles that I have found effective in guiding undergraduates to produce publishable research. These principles are largely informed by learner-centered practices (Cornelius-White, 2007) including rapport building, facilitating motivation, empowering students by honoring their ideas and opinions, encouraging problem solving, scaffolding, and internal and external self-reflection.

## Productive Labs Begin with Good Recruitment and Rapport Building

Over my time in academia, I have recruited many undergraduate research assistants (URA). I often recruit from my courses where I cover similar content to my research. I describe the types research that I engage in, as well as the benefits of working closely with a faculty member, which include fostering a close working relationship with faculty and peers, increasing confidence and knowledge, and preparation for future roles in research (Seymour et al., 2004). Best practice is to set GPA requirements and a high grade in research methods courses (Shellito et al., 2001). However, I do not share these requirements with students, as I want all students who are personally motivated by the opportunity to apply. Students are asked to submit a paragraph stating why they want to become an URA. If students do not meet the requirements but have a compelling case for how the experience with help meet their personal goals, I most often invite them to become an URA.

Despite the processed described above, at times I have inadvertently recruited students who “just need credit” and may not possess the intrinsic motivation to engage in research as a means to an end of a larger personal goal. One of most important lessons that I have learned from this is how to increase motivation. For example, positive faculty attitudes and behaviors can promote a culture of excellence in undergraduate settings (Umbach and Wawrzynski, 2005). Building rapport and a relationship with students is one of the most powerful influences on motivation, as well as the cognitive and emotional development of students (Umbach and Wawrzynski, 2005; Shanahan et al., 2015). Being approachable, respectful, and friendly have all been shown to increase students' intrinsic and extrinsic motivation (Komarraju et al., 2010) and allow students to safely explore their ideas and interests. In my lab, I make every effort to get to know my students as individuals and convey genuine interest and concern about their lives (Shellito et al., 2001; Behar-Horenstein et al., 2010). For example, during lab meeting I talk about my family and ask students about their families, their classes or other topics of interest. These informal conversations provide a window into their emotional state, stressors they may be experiencing, and specific learning challenges or strengths (Shanahan et al., 2015). Developing a deeper relationship also sets the stage for mentees who are more likely to commit to engaging in the process of publishing research beyond their semester-long laboratory experience. This is a key factor in mentoring students through publishable research and increasing faculty productivity, as most publishable works take longer than a semester to complete (Cooley et al., 2008). Finally, strong rapport increases the likelihood that students will recruit their peers to join the lab in the future, which is a helpful recruitment tool.

## Facilitating Structure, Communication and Scaffolded Expectations

After recruiting motivated students, the onus is on the faculty mentor to structure a laboratory environment that is organized, sets a standard for clear communication, and identifies expectations for the student (Mabrouk, 2003). The National Mentoring Research Network (e.g., Vishwanatha et al., 2016) suggests using “compacts” which are syllabi-like document that identify the laboratory rules and expectations. I often use a compact that includes projects for the semester, expectations for professionalism, time commitments, and how to problem solve issues (see Appendix 1). As a lab, we update this document as significant research tasks arise. I also review the compact individually with students at three points in the semester to track progress toward personalized goals. Regular review also helps me to match tasks with the students best equipped and motivated to complete them in a thorough manner.

Clear expectations of the work to be performed *between* meetings also helps to facilitate productivity and well scaffolded activity (Shanahan et al., 2015). In my laboratory, I utilize shared Google drive to do lists that are updated weekly. This structure is useful in ensuring that students know who is assigned to a task during the week, which increases workflow momentum. At each lab meeting we review the tasks on the to do list, and I allow students to choose new activities of interest to them. Students are then required to update the to do list with the status of the task throughout the week.

To facilitate a sense of community, I pair students into working groups of two and ask them to work on specific tasks together so that they are accountable to another person and can co-problem solve any issues that arise (Shanahan et al., 2015). I regularly check in with individuals about whether their partner has been accountable on tasks. If students do not complete tasks assigned to them, I gently remind them that they are part of a team that is working toward a shared goal. If issues continue, I privately discuss the issue with the student to better understand any situational factors that might be impacting their work. We problem solve strategies that could help to improve productivity, such as assigning tasks with which they are comfortable and competent to complete (Shellito et al., 2001).

Mentors should also consider each student's zone of proximal development and scaffold tasks that aid to enhance development (Thiry and Laursen, 2011). Providing meaningful experiences that are linked to clear outcomes allows students to have experiences that can feel fundamentally different from traditional didactic learning. Teaching students to actively apply their knowledge to problem based learning may contribute to a shift in students' understanding of themselves as competent researchers and life-long learners who can actively apply knowledge to solve problems (Hmelo-Silver, 2004; Davis and Jones, 2017). To demonstrate, I spend time during lab meetings discussing research methodology issues that have to be solved. Allowing students the space to think and contribute their ideas to the problem solving process fosters increased mastery in active problem solving, creates team cohesion and feelings of competence, which results an elevation in the quality of work produced (Lopatto, 2003; Shanahan et al., 2015). I also consider the developmental zones of individual students and intentionally assign task leaders who will be able to scaffold higher levels of learning beyond my direct mentorship (Gilmore et al., 2015). This structure increases the likelihood that students will receive the reinforcement of concepts at multiple times throughout the week and decrease questions directed at the mentor.

## Producing Publishable-Quality Work With Undergraduates

Creating publishable-quality work takes time and effort. It is important to acknowledge that not all undergraduate students are capable of publishable work—yet. Writing is a skill that blossoms over time, and if undergraduate students came from diverse and underserved schools with limited supports for writing, we might expect that their skills will require extra time to flourish (Early and DeCosta-Smith, 2010). I often wait until the end of a research experience to assess if someone is ready and capable of engaging in the publication process. Students who are reliably working toward achieving their learning goals, show high levels of intrinsic motivation, and have future professional goals that align with research are the best candidates. As I discuss the opportunities with qualified students, I am very clear that publishing is a long and iterative process. I explain how the publication process works, ranging from the amount of effort and time that it takes to collect and analyze data, the steps of writing a manuscript, and the review process. Students are told that they will be required to have direct and substantial intellectual contributions toward the paper, which will depend on the order of authorship (Burks and Chumchal, 2009). Students who are driven to maintain a working relationship with their faculty mentor and continue to work in the lab over the course of semesters, with or without credit, are already demonstrating the first important facet to achieving publishable work—self motivation to engage in the process (Gilmore et al., 2015).

Understanding and practicing good writing is also essential to producing publishable-quality work (Guilford, 2001). Therefore, throughout the research experience I ask students read a variety of articles and apply their foundations in research methods. We also read journal articles written with former URAs so that they can see that a journal article is an achievable goal. I then scaffold a process of guided dissemination (Shanahan et al., 2015), by first asking students to create a poster abstract which will be submitted to smaller institutional or regional conferences. This helps them to think deeper about how they would communicate the conceptual work of the research lab, both in writing and in images. Finally, for students who will continue working toward publication, I tailor manuscript writing to the student's strengths. I provide clear expectations that there will be numerous revisions before submission. To help students better achieve the goals, I break up the writing into smaller sections and provide approachable examples as models. Perhaps most importantly, however, is to have patience, provide constructive feedback, and allow the student to make multiple revisions (Guilford, 2001). It is important to emphasize that publishable writing is unlike a mastery approach where assignments are completed once (Pierce and Kalkman, 2003), but rather requires continued thought and revision over time. Rewriting the sections may be quicker for the faculty mentor (Burks and Chumchal, 2009), but it does not allow the student to learn the skills of writing and can undermine their self-esteem by sending the message that “*You can't do it, so I am going to do it for you*” (Wilson and Devereux, 2014).

## The Value of Mentor Self-Reflection

Lastly, a faculty mentor's ability to successfully lead a URA through research should include both inward and outward reflection. Most faculty mentors have not received training in pedagogy, writing, zones of proximal development, or research mentorship. However, faculty are intrinsically motivated by a number of factors, including their prior experience as a student, professional agendas and alignment with the mission of their institution (Baker et al., 2015). In addition to motivational factors, faculty mentors should continually engage in outward self-reflection about how mentorship can meet the goals of both themselves and their students. Recognizing the changing social, emotional, physical and educational needs of the whole learner can result in lifelong learning, employability, and intellectual socialization, as well as an important frame of reference for why the time and energy spent on undergraduate mentorship is worthwhile (Cornelius-White, 2007; Thiry and Laursen, 2011).

It only takes one authority figure to either bolster or undermine a student's belief in themself. Whether or not the final project gets published is a relatively minor issue compared to whether students believe that they are capable of achieving their goals. It is important to remember that students will not necessarily remember what you said or what you did, but rather how you made them feel. If they feel motivated to achieve the goals that you have set out for them, faculty mentors will pave the way for engaged students who commit to the process of producing publishable-quality work.

## Author Contributions

The author confirms being the sole contributor of this work and has approved it for publication.

### Conflict of Interest Statement

The author declares that the research was conducted in the absence of any commercial or financial relationships that could be construed as a potential conflict of interest.
