# Supplementary material for: Empowering and Motivating Undergraduate Students Through the Process of Developing Publishable Research
Source: Front Psychol. 2019 May 3;10:1007. doi: 10.3389/fpsyg.2019.01007 (PMC6509154; doi:10.3389/fpsyg.2019.01007)
Supplement: Supplementary file 1 [file Data_Sheet_1.docx]

*Appendix 1. Please find an example of an undergraduate mentoring contract/compact. We have adapted it for the “Sleepy Faces Research Laboratory” for illustrative purposes. Given that every research may operationalize their goals differently, the compact should always be personalized.*

**Undergraduate Mentoring Contract: Sleepy Faces Study**

Undergraduate Mentee: Student Name

Graduate or Faculty Mentor: Professor Adams

This project outlines the parameters of our work together on the Sleepy Faces Research Project:

1. **Project Goal:** *A description of the research project(s) the mentee will work on over the course of the semester.*

- This research will examine the effects of sleepiness on the mental and physical health of college students. Objective measure of sleep will be measured, as well as self report measures of sleepiness, mental health, physical health, academic functioning, and demographic information.

2. **Why is this project important?:** *Describe the goals of the research project and what contributions it will make to the field and future research.*

- This study will provide valuable information about how to estimate sleepiness in a real world sample of college undergraduates. The study seeks to validate a method for reliably quantifying sleepiness based on unobtrusive measures obtained by videotape. If successful, this method could be used in clinical and operational settings to improve diagnoses and to prevent accidents.

3. **Research Objectives:** *Describe the specific research objectives for this semester.*

1) Explore associations among objective and subjective measures of sleepiness in college students

2) Explore the effects of inadequate sleep on multiple indicators of health and well-being, including anxiety, depression, physical health status, and academic performance,

3) We will develop a manualized protocol for scoring sleepiness based on facial displays in response to videos.

4. **Expectations for Undergraduate Mentees:** *Clearly describe your expectations for the undergraduate mentee in the lab.*

- Attend lab meetings and arrive on time. If you will be absent email, call or text in advance.
- Be organized: This means that you will come prepared to all meetings. Please read assigned journal articles in advance. Come prepared to update the group on progress toward goals. When working with a partner in the lab, come prepared with any required materials. During lab meeting, take detailed notes as Professor Adams describes the tasks to be worked on during the week.
- Read background information and protocols about our projects. Bring any questions that arise from the readings to lab meetings.
- Be independent: In our lab meeting, Professor Adams will demonstrate and explain tasks. Ask questions if you are confused. If you are working on a task and get stuck, try to problem solve by: 1) looking at your notes, 2) asking a peer for help, and 3) asking Professor Adams. Independent work requires that you try to actively solve problems before asking for an answer.
- Respect the lab area and your colleagues: Please keep the lab space neat and clean and as you found it when you first entered. Speak to your colleagues with respect - you should speak to each other as if I am present in the room. Always be accountable for your behavior and treat others how you expect to be treated.
- Ask questions: You are here to learn and I am here to teach you. Questions are always welcomed and encouraged. Please do not proceed in a task in which you are confused, as it could impact the results of our the research!

5. **Time Commitment:** *This should include the time commitment over the semester.*

- This 3-credit placement will last for 14 weeks during the Fall semester.
- It is possible that I will need help running participants during finals week. If so, we will discuss whether you will be available and the possibility of comp time.

6. **The Mentor and Mentee will be in contact by:** *Clearly outline how you would like students to contact you.*

- Phone/email/ face-to-face.
- If you are in immediate need of assistance please text at xxx-xxx-xxxx.

7. **Meeting Length:** *This should include the amount of time that students are expected to work both inside and outside of the lab.*

- We will meet one-on-one for 60 minutes 1 time per week.
- It will be the (**mentor’s**/ mentee’s) responsibility to schedule these meetings.
- In preparation for these meetings the mentee will: complete the work the mentor has assigned the week before including all readings and tasks.
- In preparation for these meetings the mentor will: organize work for the mentee to complete for the following week, identify training needs, and coordinate resources needed to accomplish tasks for the week.
- At these meetings, the mentor will provide the mentee specific feedback for how to improve or progress through verbal evaluation.
- All students will be required to complete a weekly time log on Google Sheets.

8. **The mentee will learn new techniques and procedures by:**

- Meeting with principal investigator to receive training in research methodology.
- Meeting with the graduate student who will model research methodology and help to clarify and reinforce initial training.
- It is expected that students will practice laboratory techniques on other mentees prior to working with research participants.

9. **If the mentee gets stuck while working on the project:**

- Contact peers, grad student, and principal investigator directly via text or phone.

10. **The standard operating procedures for working in our research group, which all members must follow and agree to are:**

- Communicating with team members about device malfunctions, restocking materials, actigraph pick up, and cancellations.
- Being mindful of our workspace in the shared research laboratory and cleaning the space after each use. All materials should be returned to the locked file cabinet.
- At the end of each work session, each student must update the **Shared Task Log** with a) where they left off in tasks, and b) any comments for the next student to work on the task.

11. **Other issues not addressed above that are important to our work together:**

- At the end of the semester, the mentee will complete a final project to receive credit for the research placement.
- In an effort to match the project to the student’s interest areas, this project will be determined in conjunction with the student.
- At times there are opportunities for students to engage in writing projects that could be published at a later date. These projects require students to work with Professor Adams for a year or two (or more) beyond this semester. If interested, we will discuss opportunities for publishing.

***By signing below, we agree to these goals, expectations, and working parameters for this research project.***

Mentor signature ________________________________________________

Mentee Signature _______________________________________________
